# Revealing the therapeutic potential of synthetic cannabinoids: a systematic review of cannabinoid receptor binding dynamics and their implications for cancer therapy

**DOI:** 10.1186/s42238-025-00289-5

**Published:** 2025-06-07

**Authors:** Sahar S. Alghamdi, Hussah N. Albahlal, Danah E. Aloumi, Sarah Bin Saqyah, Arwa Alsubait, Jehan Alamre, Mohammed Alrashed, Nada Alsuhabeny, Afrah E. Mohammed

**Affiliations:** 1 Department of Pharmaceutical Sciences, College of Pharmacy, King Saud Bin Abdulaziz University for Health Sciences, 11451 Riyadh, Saudi Arabia; 2 Medical Research Core Facility and Platforms, King Abdullah International Medical Research Center (KAIMRC), Ministry of National Guards Health Affairs, Riyadh, Saudi Arabia; 3 Department of Clinical Laboratory Sciences, College of Applied Medical Sciences, King Saud Bin Abdulaziz University for Health Sciences, Riyadh, Kingdom of Saudi Arabia; 4 King Abdulaziz Medical City, National Guard Health Affairs (NGHA), Riyadh, Saudi Arabia; 5 Department of Biology, Faculty of Science, Princess Nourah Bint Abdulrahman University, Riyadh, Saudi Arabia; 6 Microbiology and Immunology Unit, Natural and Health Sciences Research Center, Princess Nourah Bint Abdulrahman University, Riyadh, Saudi Arabia

**Keywords:** CB1 Receptor, CB2 Receptor, Synthetic Cannabinoid, Cancer, Agonist, Antagonist

## Abstract

**Background:**

Cancer remains a major global health issue, prompting the need for innovative treatment approaches that extend beyond conventional methods such as chemotherapy and radiation. The endocannabinoid system (ECS), primarily the cannabinoid receptors CB1R and CB2R, presents a promising opportunity for cancer therapy by selectively targeting cell signaling pathways. This systematic review intends to explore the mode of action of synthetic cannabinoids as potential anticancer agents and their impact on tumor growth in various cancer cell lines.

**Methods:**

Of the 287 articles identified between January 1990 and July 2024, 27 studies met strict criteria focusing on their anticancer effects. Data extraction and quality assessment were conducted using GRADE criteria and the Cochrane Risk of Bias tool, ensuring robust evaluation of the studies' reliability.

**Results:**

Various pharmacological actions of synthetic cannabinoids function as agonists, antagonists, and inverse agonists at the CB1R and CB2R receptors. Key findings indicate that CB2R agonists significantly reduce cancer cell proliferation through diverse mechanisms, with selective CB2R agonists effectively inhibiting cancer cell growth and survival. Studies involving CB1R antagonists, particularly in conjunction with CB2R agonists, highlight their role in blocking CB1R to either validate or enhance the efficacy of CB2R agonists in mitigating tumor growth. Inverse agonists targeting CB2R have shown moderate success in inducing cancer cell death by disrupting survival pathways. Notably, synthetic cannabinoid agonists display significant potential in targeting CB1 and CB2 receptors to inhibit tumor proliferation and promote apoptosis across various cancer types.

**Conclusion:**

The systematic review concludes that CB2R agonists can effectively inhibit tumor growth while inducing apoptosis in various cancers. Although CB1R agonists show potential in modulating cancer pathways, there is a notable lack of research on CB1 inverse agonists, emphasizing the need for further investigation. Additionally, the study advocates for greater exploration of mixed receptor agonist and receptor mode of action to validate these promising therapeutic approaches.

**Graphical Abstract:**

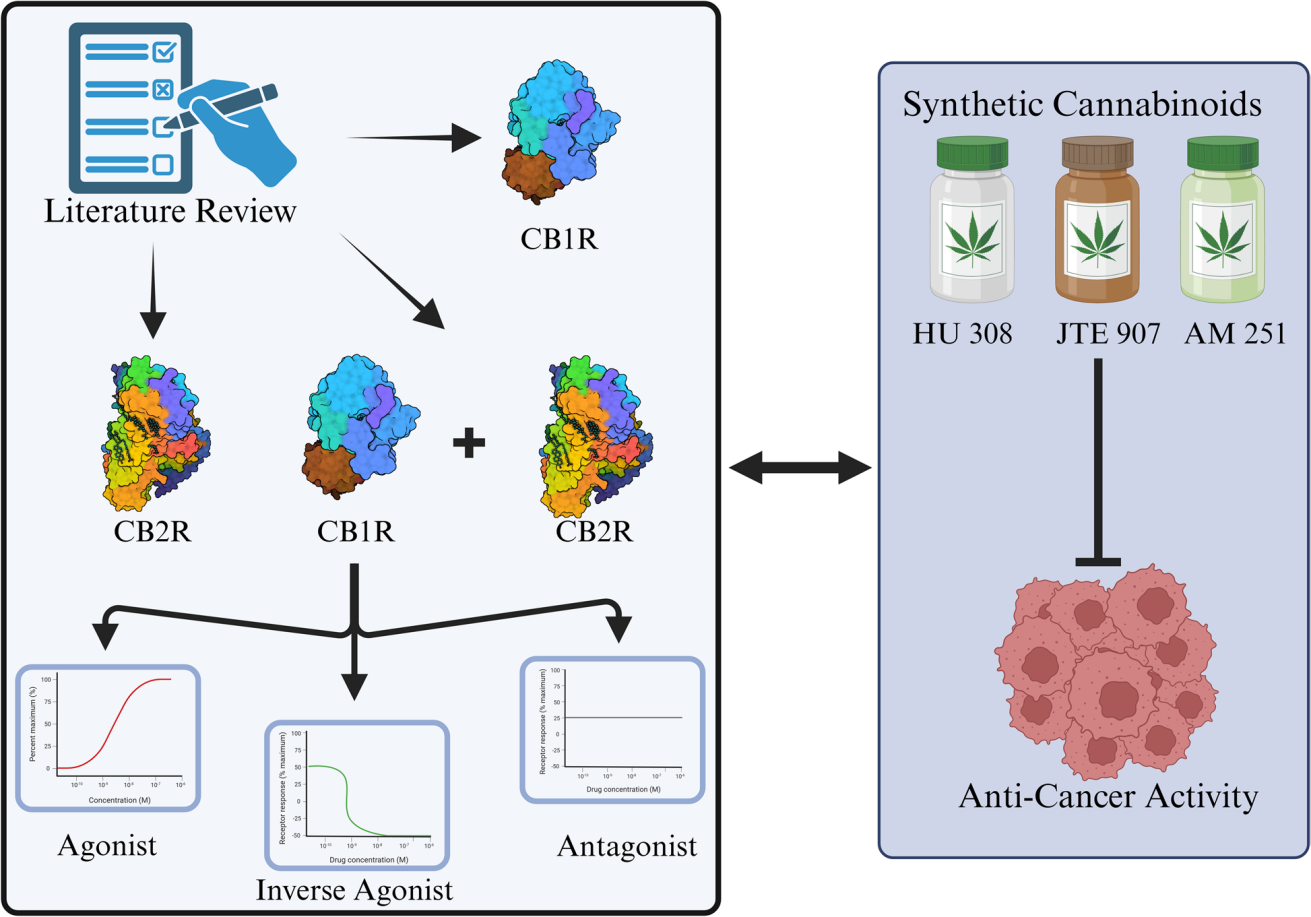

## Introduction

**Fig. 1:**
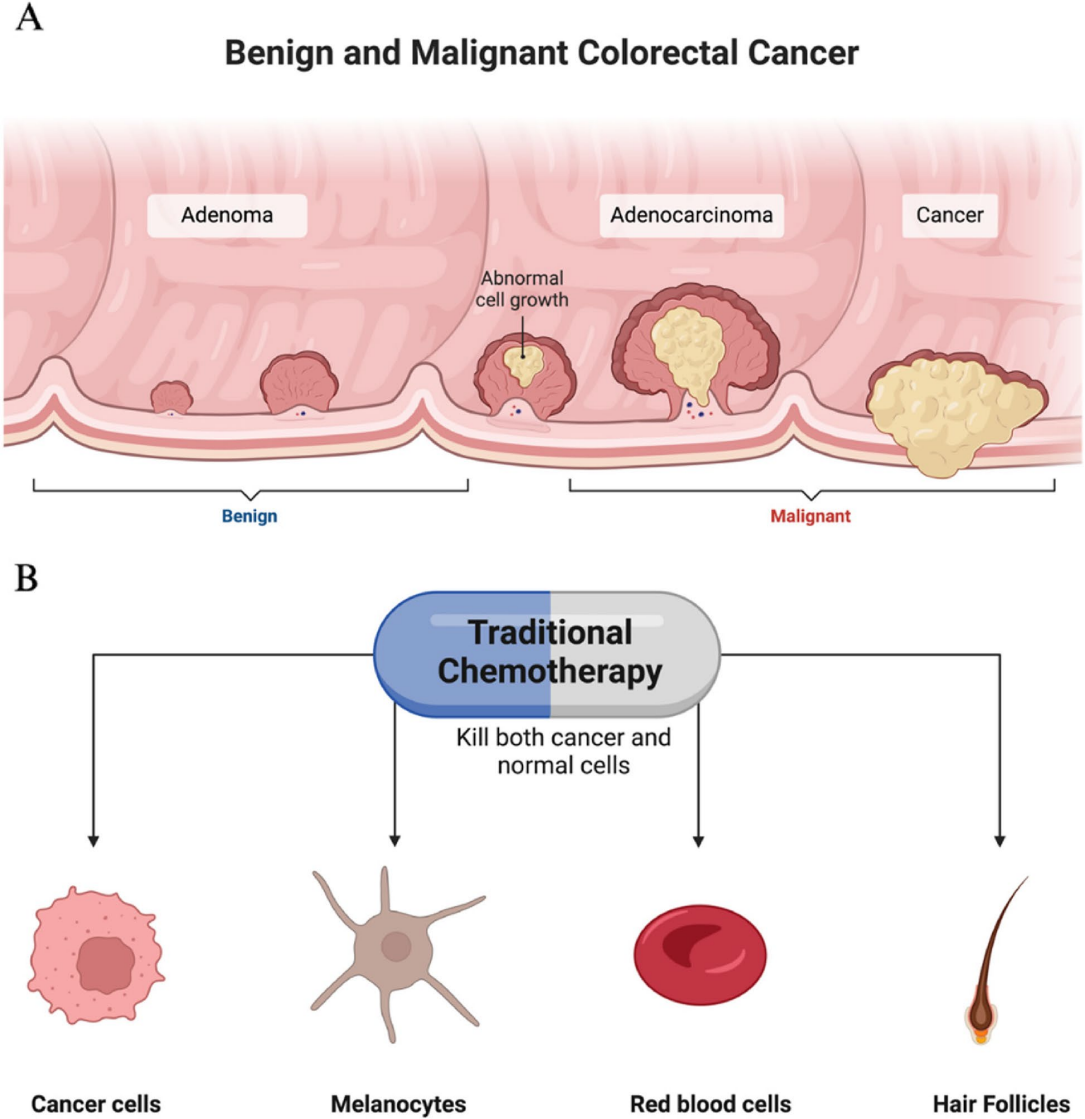
**A** Progression of colorectal cancer from benign to malignant stages. This illustration shows the development of colorectal cancer, beginning with benign growths (adenomas) that can, over time, progress to adenocarcinoma and eventually to malignant cancer. **B** Non-selective targeting of chemotherapy on cancer and healthy cells. This illustration highlights the limitations of traditional chemotherapy, which affects not only cancer cells but also healthy cells such as melanocytes, red blood cells, and hair follicles. This lack of specificity contributes to the adverse side effects associated with chemotherapy, highlighting the need for more targeted therapeutic approaches in oncology

Cancer is a complex disease characterized by the abnormal growth of different types of cells in the body. There are over a hundred distinct types of cancer, each with different behaviors and responses to treatment. It is important to distinguish between benign and malignant tumors in cancer pathology (Jones 2023), as illustrated in Fig. 1. In the World Health Organization (WHO) Eastern Mediterranean Region alone, over 788 000 individuals were diagnosed with cancer in 2022. This number is anticipated to treble to reach 1.57 million cases by 2045, due to population increase and, more crucially, the high prevalence of cancer risk factors in the Region—such as tobacco use, obesity/overweight, physical inactivity, poor diets, and air pollution (Cancer, 2025; Cancer Tomorrow, 2025). In Saudi Arabia, the incidence of various cancers has tripled in recent years, likely due to lifestyle changes towards a Western model, limited cancer awareness, and inadequate screening programs. Key risk factors include obesity, genetics, sedentary habits, tobacco use, viral infections, and deficiencies in iodine and vitamin D (Ahmed et al. 2024). The selective targeting of cancer cells remains a significant challenge in oncology, hindering the effectiveness of current therapeutic strategies. Therefore, there has been a growing emphasis on alternative treatments targeting molecular pathways involved in cancer formation and progression. A promising avenue for anticancer research lies in the recent discovery of cannabinoid receptor ligands (Walsh, 2020).

**Fig. 2:**
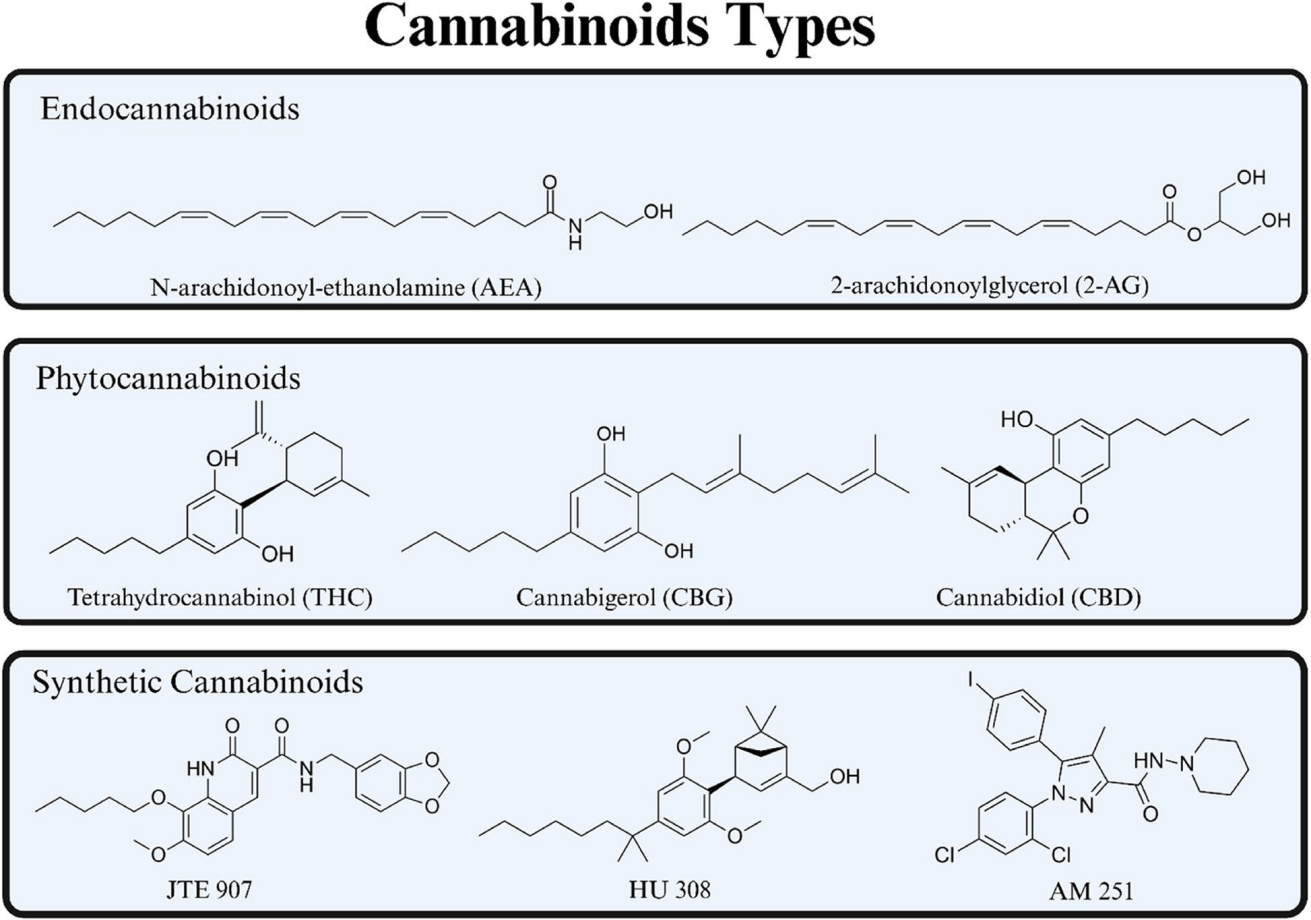
Types of cannabinoids and representative structures. This figure illustrates the three types of cannabinoids: endocannabinoids, AEA and 2-AG, which are naturally produced in the body; phytocannabinoids, CBD, CBG, and THC, from Cannabis sativa; and synthetic cannabinoids, AM 251, HU 308, and JTE 907, which are lab-made for research and therapy. Each type interacts uniquely with the endocannabinoid system

**Fig. 3:**
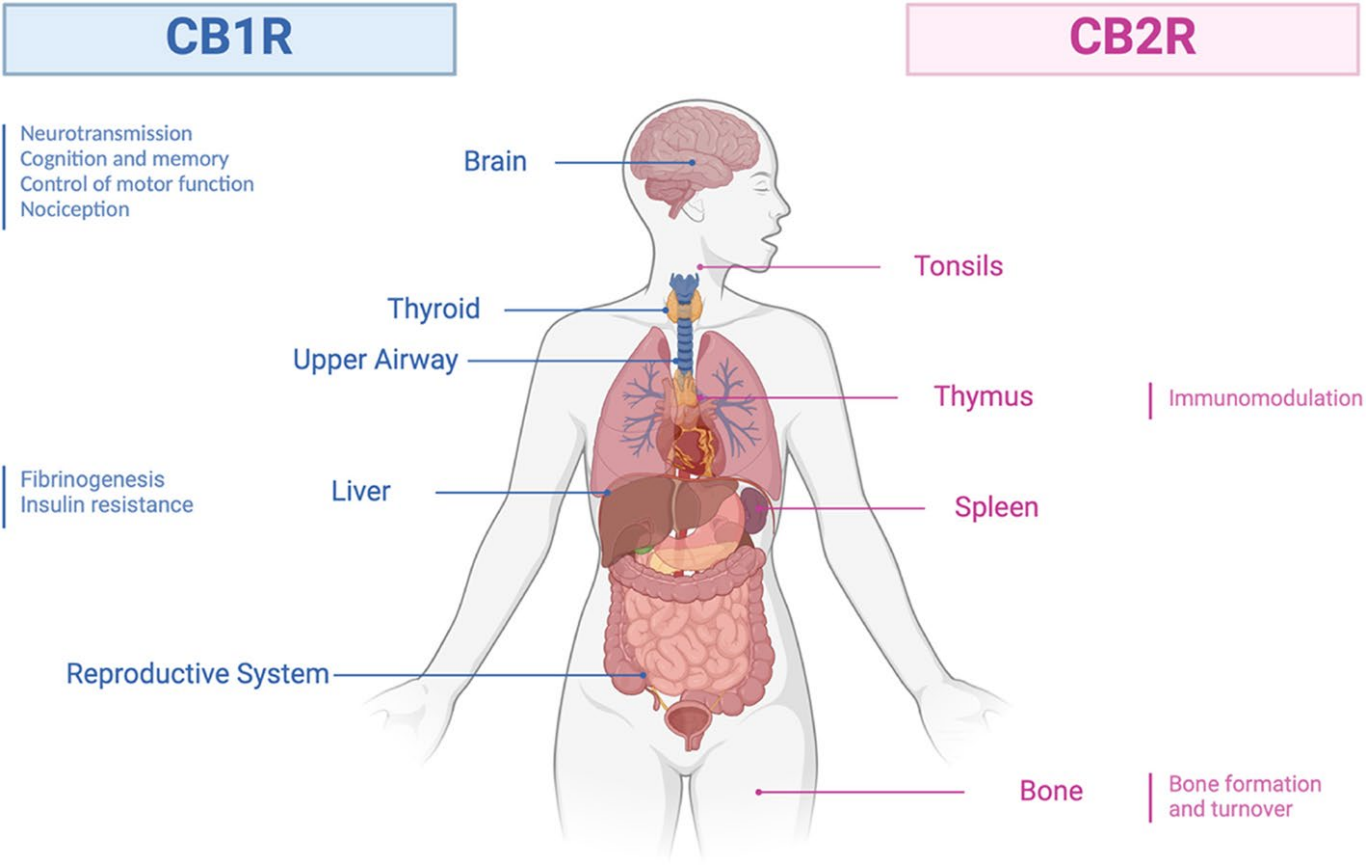
Localization and functions of cannabinoid receptors CB1R and CB2R. CB1R is primarily located in the brain, influencing neurotransmission, cognition, and pain perception. It is also present in the thyroid, liver, and reproductive system, affecting metabolism and insulin resistance. CB2R is mainly found in immune organs like the tonsils, thymus, and spleen, playing a key role in immune modulation and in bones, where it supports bone turnover. These distinct localizations highlight CB1R's neurological roles and CB2R's immune functions, making them important therapeutic targets

The endocannabinoid system (ECS) is a complex biological network composed of endogenous lipid-based retrograde neurotransmitters called endocannabinoids, cannabinoid receptors (CBRs), and the metabolic enzymes responsible for synthesizing and degrading endocannabinoids (Lu & Mackie 2021). N-arachidonoyl-ethanolamine (AEA) and 2-arachidonoylglycerol (2-AG) are a major endocannabinoids in vivo system, as illustrated in Fig. 2 (Lu & Mackie 2016). Cannabinoid receptor 1 (CB1R) and cannabinoid receptor 2 (CB2R), components of ECS, are G protein-coupled receptors primarily coupling to inhibitory G proteins (Moreno et al., 2019). CB1R is primarily found in the central nervous system, while CB2R is predominantly found in the peripheral system, particularly in immune cells (Bie et al. 2018), as shown in Fig. 3. CB2R plays a crucial role in inflammation, immunity, and cancer progression (Hashiesh et al. 2021). This comprehensive system helps maintain homeostasis and regulates a wide range of physiological and pathological processes, including tumorigenesis and tumor suppression. Although the therapeutic potential of this intricate ECS network is promising, the direct anticancer properties of ligands targeting CBRs remain largely unexplored. Further investigation is needed to understand their potential as direct anticancer agents rather than as modulators of associated physiological processes.

Phytocannabinoids, which are the natural cannabinoids found in *Cannabis sativa*, have been extensively studied for their potential anticancer effects (Koltai & Shalev 2022). These compounds act as agonists for cannabinoid receptor 1 and cannabinoid receptor 2, facilitating their therapeutic applications through the activation of these CBRs (Gülck & Møller 2020). By activating CB1R and CB2R, phytocannabinoids produce various therapeutic effects, including anti-nociception, anti-inflammation, anticonvulsant, and anti-emetic properties (Zou & Kumar 2018). However, the recreational use of these compounds has predominantly limited their medical applications. An example of a phytocannabinoid is tetrahydrocannabinol (THC), as shown in Fig. 2, which acts as an agonist at CBRs and exhibits similar pharmacological effects to endocannabinoids (Maccarrone & Finazzi-Agró, 2003).

Many newly synthesized cannabinoid analogs demonstrate high specificity toward CB2R. HU308 is a synthetic compound with agonistic behavior and selectivity for CB2R (Hanuš et al. 1999). Furthermore, HU308 causes extracellular-regulated kinase (ERK_1/2_) and p38 to be down-regulated, and it also reduces the production of IL-6 and matrix metalloproteinase, specifically MMP-3 and MMP-13, in IL1β-activated fibroblast-like synoviocytes that are generated from synovial tissue from rheumatoid arthritis (Gui et al. 2014). Nevertheless, research on HU308's impact on anti-neoplastic activity and the mechanisms regulating it remained lacking.

Similarly, a recent investigation was conducted on the use of AM630 as a CB1R/CB2R antagonist in non-small cell lung cancer (NSCLC). According to the study, the agonist-mediated suppression of in vitro chemotaxis and chemoinvasion was reduced when a CB1R/CB2R-specific antagonist was administered (Preet et al., 2011). On the other hand, JTE 907 is an extremely specific inverse agonist of CB2R. It has anti-inflammatory properties in vivo and binds to CB2 receptors in rats, mice, and humans with high-affinity K_i_ values of 0.38, 1.55, and 35.9 nM, respectively (Iwamura H et al. 2001).

Despite the clinical use of synthetic cannabinoids for several years, their precise molecular mechanism of action remains poorly understood (Hinz & Ramer 2022). Most research to date has focused on investigating the hypothesis that these compounds possess anti-tumor properties (Voicu et al. 2023). In this comprehensive systematic review, we aim to address this research gap by methodically examining the mode of action of synthetic cannabinoid ligands as agonists, antagonists, and inverse agonists and their modulation of the CB1R and CB2R. Ultimately, we will investigate the impact of the ligand mode of action of synthetic cannabinoids on tumor growth across various cancer cell lines. This analysis will provide critical insights into the potential anticancer effects of these compounds and the underlying signaling pathways involved.

## Methodology

### Data sources and search strategy

A comprehensive literature search was conducted across PubMed, Science Direct, Google Scholar, and the Cochrane Database to identify relevant studies published from January 1990 to July 2024. The search strategy employed a combination of Medical Subject Headings (MeSH) terms and keywords related to synthetic cannabinoids and cancer. The search terms included"cancer,""anticancer,""neoplasm,""antineoplasm,""tumor,""antitumor,""synthetic molecules,""agonist,""antagonist,""inverse agonist,""mixed agonist/antagonist,""cannabinoid receptor 1,"and"cannabinoid receptor 2."Boolean operators"AND"and"OR"were used to combine terms effectively (e.g.,"synthetic cannabinoids"AND"anticancer"AND"cannabinoid receptor 1"). Reference lists of pertinent articles were also reviewed to ensure the inclusion of all relevant studies.

### Study selection and eligibility criteria

The initial search yielded 287 articles, screened by two independent reviewers using the predefined inclusion and exclusion criteria. To be eligible for inclusion, studies needed to report on the anticancer effects of synthetic cannabinoid receptor ligands, be conducted as in vitro or in vivo experiments, be published between January 1990 and July 2024, be available in full text and published in English, and focus specifically on the mode of action of synthetic cannabinoid ligands and their anticancer activity. Studies were excluded if they were review articles, systematic reviews, unpublished articles, dissertations, commentaries, conference proceedings, case reports, book reviews, opinion articles, or editorials. Additionally, articles not accessible in full text, published in languages other than English, not specifically focused on cancer, or not involving synthetic cannabinoids as defined in the study parameters were excluded. Discrepancies between the reviewers were resolved through discussion or consultation with a third reviewer when necessary. After applying the inclusion and exclusion criteria, four studies were selected for detailed analysis.

### Data extraction and quality assessment

Three reviewers independently extracted data using a standardized form. The information extracted included the authorship, publication year, study design, type of synthetic cannabinoid receptor ligand, type of cancer investigated, experimental models used (in vitro and in vivo), key findings on anticancer effects, and details regarding each ligand’s mode of action at cannabinoid receptors.

The quality of the included studies was assessed using the Grading of Recommendations Assessment, Development, and Evaluation (GRADE) criteria, adapted for preclinical research. Each study was evaluated for risk of bias, directness of evidence, consistency of results, precision, and publication bias. The overall quality of evidence was categorized as high, moderate, low, or very low. Any disagreements in data extraction or quality assessment were reconciled through consensus or consultation with a third reviewer to ensure accuracy and reliability. All studies were evaluated for potential bias by two independent researchers. The Cochrane Risk of Bias (ROB) assessment tool was used to assess the overall risk of bias in the randomized controlled trials included in the review.

## Results

**Fig. 4:**
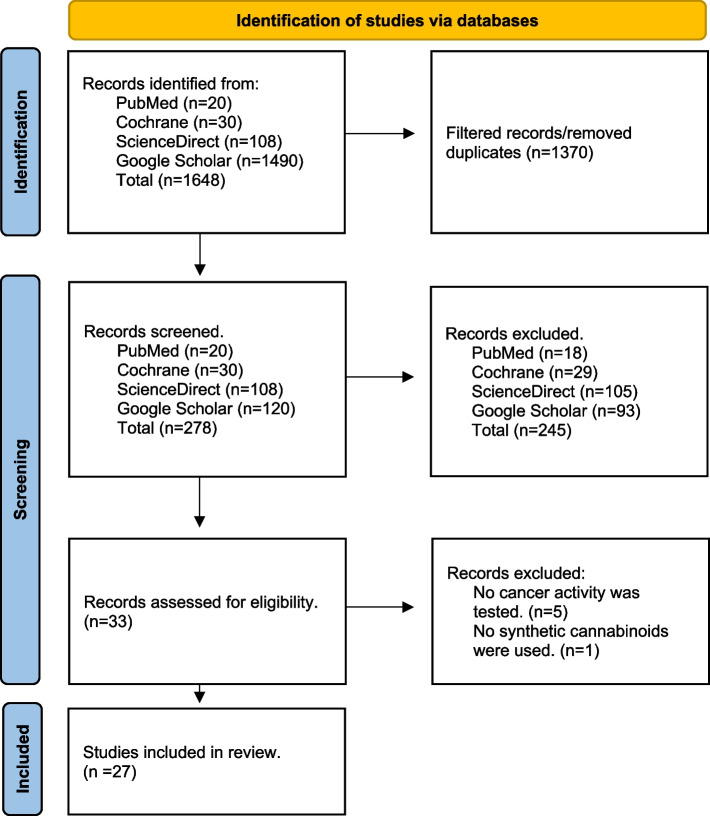
Illustrate the search strategy to include 27 of 278 screened articles using the PRISMA diagram

After a comprehensive literature search, 287 articles were screened, but only 27 were included in this systematic review, as shown in Fig. 4. These studies primarily investigated the impact of synthetic cannabinoids on tumor progression and examined how their mode of action at cannabinoid receptors contributes to the inhibition of tumor growth.

Synthetic cannabinoids can act as agonists, antagonists, or inverse agonists at CB1 and CB2 receptors, leading to diverse pharmacological effects across cancer cell lines. This section explores the anticancer activity of these compounds, with emphasis on their mode of action at cannabinoid receptors.

### Synthetic cannabinoids 1 receptor

#### CB1R antagonists/inverse agonist

Our comprehensive review of CB1R antagonists and inverse agonists revealed diverse effects across various cancer types. As the CB1R inverse agonists showed their ability to reduce CB1R basal activity and mimic the antagonism mode of action, this section combined these two modes of action acting on CB1R. The findings are organized by cancer type, highlighting the key outcomes and mechanisms observed in each case illustrated in Table 1.

**Table 1:** Summary of studies investigating CB1R ligands in cancer cell apoptosis, proliferation, and metastasis

Study	Ligand Used	Cancer Type	Cell Line (s)	Mechanism of Action	Key Outcome
CB1R Antagonists					
(Aviello et al. 2012)	SR141716 (Rimonabant) and AM251	Human colon cancer cell lines	HCT116 and Caco-2 cell lines	Combination between CB1R antagonist and cannabidiol	Antiproliferative effects in vitro via CB1R activation
(Preet et al., 2011)	AM251	Non-small cell lung cancer	A549 and SW-1573 cell lines	Tumour growth and metastasis	Inhibited non-small cell lung cancer cell proliferation, migration, and invasion both in vitro and in vivo
(Qamri et al., 2009)	AM 251	Primary human breast cancer	MDA-MB231, MDA-MB231-luc, and MDA-MB468 cell lines	Validation in vivo for many mouse model systems by reversed the CB1and 2 agonists’ effects to ensure that the cancer reduction is mediated by the effect of CB1 and 2 receptor agonists only	Validation that CB1 and CB2 agonists are responsible of 40% to 50% reduction in tumour development and a 65% to 80% reduction in lung metastasis
(Raup-Konsavage et al. 2018)	PTI-1, PTI-2, and NPB-22	Colorectal cancer	SW480, SW620, HT29, DLD-1, HCT115, LS174, and RKO cell lines	PTI-1and PTI-2: decreased the vitality of all cell lines examined, including HEK293 and CCD841 CoTr (normal cells) NPB-22: No known mechanisms	↓cancer progression
(Fogli et al. 2006)	AM251and SR141716 A	Human pancreatic cancerous cells	MIA PaCa-2 cancer cell line	SR141716 A: No significant impact on cell viability AM251: alters the transcription of genes in janus kinase/signal transducers and activators of the transcription signalling network, and interacts synergistically with 5-fluorouracil, a pyrimidine analogue	SR141716 A: No effect on cancer cell lines AM251: trigger apoptosis
(Widmer et al. 2008)	AM251	Glioma cancer cells	U373MG cell linem	Prevent cannabinoid-induced cell death in normal cell lines	No cancer effect on cancerous cell by itself, but it has protective role for normal cell lines
(Sarnataro et al. 2006)	SR141716 (Rimonabant)	Human breast cancer cell	MDA-MB-231 cells	Antiproliferative activity of SR141716 is mediated by the cannabinoid CB1R activation	↓the growth of breast cancer and hinder metastatic diffusion
(Blázquez et al. 2006)	SR141716 (Rimonabant)	Melanoma cell lines	B16 and A375	CB1R inhibitions	No significant effect of SR141716 (Rimonabant) on melanoma
(K. Gustafsson et al. 2009)	SR141716 A	Mantle cell lymphoma	They study did not specify the cell line	Blockage the CB1-mediated signalling pathway that leads to the activation of the de novo ceramide production pathway, which includes the enzymes CerS3 and CerS6	Cancerous cell death
(Malfitano et al. 2012)	SR141716 (Rimonabant)	Fibrosarcoma cell line	Methylcholanthrene-induced fibrosarcoma (Meth-A) cell line	Reduced COX2 expression and induction of the CB1R expression	↓cancer spread via inducing apoptosis
CB1R Agonist					
(Gurley et al., 2012)	KM-233	Glioblastoma multiforme	U87MG	modulating protein phosphorylation	Cellular signalling pathways alteration

##### Colon cancer

An in vitro study on human colon cancer cell lines (HCT116 and Caco-2) showed that CB1R antagonists, particularly Rimonabant (SR141716) and AM251, may contribute to anticancer properties. The mechanism involves indirect activation of CB1 receptors, resulting in antiproliferative effects (Aviello et al. 2012).

Another study evaluated a synthetic cannabinoid library on seven colorectal cancer (CRC) cell lines: SW480, SW620, HT29, DLD-1, HCT115, LS174, and RKO. The PTI family of compounds decreased the vitality of all cell lines examined, including normal cells (HEK293 and CCD841 CoTr). NPB-22 showed efficacy in only two cell lines (HCT116 and SW480) through an unknown mechanism (Raup-Konsavage et al. 2018).

AM251, a selective CB1R antagonist, demonstrated a protective effect on human colorectal carcinoma Caco-2 cells in vitro. Both CB1 and CB2R antagonists reduced the cytotoxic effects of HU 210 and AEA (S. B. Gustafsson et al., 2008).

##### Lung cancer

Research on non-small cell lung cancer (A549 and SW-1573 cells) demonstrated that synthetic cannabinoid agents, including CB1/CB2 agonists WIN 55,212–2 and JWH-015, significantly decreased the focal adhesion complex crucial for cancer cell migration. Pretreatment with AM251 (CB1 antagonist) and AM630 (CB2 antagonist) confirmed that the cancer-reducing effect was primarily mediated by cannabinoid receptor agonists. The main outcome was inhibition of cell proliferation, migration, and invasion both in vitro and in vivo (Preet et al., 2011).

##### Breast cancer

Studies on primary human breast cancers and breast cancer cell lines (MDA-MB231, MDA-MB231-luc, and MDA-MB468) revealed expression of CB1 and CB2 receptors. Synthetic CB2 agonists and CB1/2 agonists reduced cell growth and migration in vitro*. *In vivo studies in mouse models showed a 40–50% reduction in tumor development and a 65–80% reduction in lung metastasis with CB1 and CB2R agonist treatment. The CB1 antagonist AM251 reversed these effects, confirming the role of CB1 receptors in cancer development reduction (Qamri et al., 2009).

An in vivo study on MDA-MB-231 cells (more invasive than T47D and MCF-7 cells) demonstrated that the CB1R mediates SR141716’s antiproliferative activity. The effect resulted from a G1/S-phase cell cycle arrest rather than apoptosis or necrosis (Sarnataro et al. 2006).

##### Pancreatic cancer

An in vitro study on human pancreatic cancer cells (MIA PaCa-2) using AM251, SR141716 A, and 5-Fluorouracil showed that AM251 triggered apoptosis and altered gene transcription in the Janus kinase/signal transducers and activators of transcription (JAK/STAT) signaling pathway. AM251 also demonstrated synergistic effects with 5-fluorouracil. However, WIN-55,212–2 (non-selective cannabinoid receptor agonist) and SR141716 A did not significantly impact cell viability at 10 μM over 24–72 h (Fogli et al. 2006).

##### Glioma

Investigations on U373MG glioma cells revealed that AM251 almost completely inhibited cannabinoid-induced cell death in normal cell lines, suggesting a crucial role for CB1 receptors in cannabinoid-triggered apoptosis (Widmer et al. 2008).

Another study on U87, U373, U118, and A172 human glioma cells showed no significant effect of SR141716a on cancer spread when used as a pretreatment (Recht et al. 2001).

Research on C6 glioma cells demonstrated that blocking both CB1 (SR141716 A) and CB2 (SR144528) receptors was necessary to protect cells from the antiproliferative effects of Δ9-THC (Vaccani et al. 2005).

##### Melanoma

In vitro studies on melanoma cell lines (B16 and A375) concluded that SR141716 (Rimonabant) had no significant effect on melanoma. The antagonist was primarily used to investigate the impact of cannabinoid receptors on melanoma cell lines (Blázquez et al. 2006).

##### Lymphoma

Research on Mantle Cell Lymphoma showed that SR141716 A blocks the CB1-mediated signaling pathway, leading to the activation of the de novo ceramide production pathway (involving enzymes CerS3 and CerS6) and resulting in cell death (K. Gustafsson et al. 2009).

##### Fibrosarcoma

An in vitro study on methylcholanthrene-induced fibrosarcoma (Meth-A) cell line revealed that Rimonabant (SR141716) reduces cancer spread by inducing apoptosis. The mechanism involves modulating cell cycle progression through regulation of p21waf, cyclins E, D1, and NF-κB (Malfitano et al. 2012).

In conclusion, CB1R antagonists demonstrate varied effects across different cancer types, ranging from antiproliferative and pro-apoptotic activities to protective effects in some cases. These findings underscore the complex role of the endocannabinoid system in cancer biology and highlight the need for further research to fully elucidate the potential of CB1R antagonists in cancer treatment.

#### CB1R agonist

A single synthetic cannabinoid agent that is categorized under CB 1 receptor agonists was studied on human glioblastoma multiforme (GBM) cell line U87MG via in vivo* and *in vitro settings. Cellular signalling pathways alteration was mediated by KM-233 modulating protein phosphorylation in a time-dependent manner, including MEK, ERK1/2, Akt, BAD, STAT3, and p70S6 K (Gurley et al., 2012). No further studies examined the effect of synthetic cannabinoid agonists on CB1 receptors in cancer development. Table 1 outlines the effects of synthetic cannabinoids from CB1R agonists on cancer cells, involving apoptosis, proliferation, and metastasis.

### Synthetic cannabinoids 2 receptor

#### CB2R antagonist

##### Breast Cancer

SR144528, a CB2R-specific antagonist, has been extensively used in studies to clarify the role of CB2 receptors in mediating the antiproliferative and antimetastatic effects of synthetic cannabinoids on cancer cells. Another study evaluated the synthetic CB2R agonist JWH-133 in breast cancer. In the study by (Qamri et al. 2009), JWH-133 inhibited the proliferation and migration of breast cancer cell lines. These effects were reversed when SR144528 was applied, confirming the CB2R-dependent nature of the response. In vivo experiments further demonstrated that JWH-133’s reduction in tumour growth and metastasis was negated by SR144528, underscoring its role in blocking CB2R-mediated antitumor effects.

Similarly, (Blázquez et al. 2006) study reinforced SR144528's ability to block CB2R-mediated actions. It reversed the anti-proliferative and anti-migratory effects of JWH-133 on breast cancer cells, which prominently expressed CB2Rs, such as MDA-MB231 and MDA-MB468. Moreover, MDA-MB231 cells treated with JWH-133 exhibited a 46% reduction in tumor growth, but this was reversed by SR144528. These findings highlight the significance of CB2Rs in regulating the therapeutic effects of cannabinoids and suggest that blocking CB2R can diminish these effects.

##### Glioma

In (Recht et al. 2001) study on the antitumor effects of ajulemic acid (AJA), another synthetic cannabinoid, CB2R antagonism was crucial in understanding its mechanism of action. AJA demonstrated significant antitumor activity, primarily mediated through CB2Rs. The CB2R antagonist SR144528 reversed these effects, particularly in C6 glioma cells, where co-incubation led to a 63% reduction in AJA’s inhibitory action on tumour growth. This contrasted with THC, which was blocked by both CB1R and CB2R antagonists, further confirming that AJA’s antitumor efficacy is predominantly reliant on CB2 receptors.

Additionally, the study by (Jacobsson et al., 2001) examined the role of CB2R in cancer cell proliferation using JWH-015, a selective CB2R agonist. In C6 glioma cells, JWH-015 significantly inhibited proliferation, an effect that was reversed by SR144528. This reinforced the idea that CB2R activation is crucial for the antiproliferative effects of synthetic cannabinoids and that SR144528 serves as a reliable tool in verifying CB2R dependency in these responses.

##### Colorectal cancer

In contrast, (Aviello et al. 2012) study on the chemopreventive effects of cannabidiol (CBD) in colorectal cancer cells revealed that CB2R activation was not a primary pathway. In human colorectal carcinoma cell lines Caco-2 and HCT116, the CB2R antagonists SR144528 and AM630 did not significantly alter the antiproliferative effects of CBD. However, the effects were reversed by CB1R antagonists, suggesting that the CB1R, rather than CB2R, plays a more critical role in mediating CBD’s anticancer effects. seen in Table 2.

**Table 2:** Summary of studies investigating CB2R ligands in cancer cell apoptosis, proliferation, and metastasis

Study	Ligand Used	Cancer Type	Cell Line (s)	Mechanism of Action	Key Outcome
CB2R Antagonists					
(Qamri et al., 2009)	SR144528	Breast cancer	MDA-MB231, MDA-MB468 cell line	Inhibited CB2 receptor-mediated pathways, blocking antiproliferative and anti-migratory signals	Blocked antiproliferative and anti-migratory effects of JWH-133
(Blázquez et al. 2006)	SR144528	Breast cancer	MDA-MB231 cell line	Antagonized CB2R signaling, reversing the tumor growth reduction induced by JWH-133	Reversed the 46% reduction in tumor growth induced by JWH-133
(Recht et al. 2001)	SR144528	Glioma	C6 Glioma cell line	Suppressed CB2 receptor-mediated antitumor pathways, reversing antitumor pathways of Ajulemic Acid (AJA)	Reversed 63% of Ajulemic Acid’s (AJA) antitumor effects
(Jacobsson et al., 2001)	SR144528	Glioma	C6 Glioma cell line	Reversed CB2 receptor-mediated antiproliferative effects induced by JWH-015	Reversed JWH-015’s antiproliferative effects
(Aviello et al. 2012)	SR144528	Colorectal Carcinoma	Caco-2 and HCT116	CB2R plays a minimal role in CBD’s anticancer effects, with its blockade showing negligible impact	CB2R is less critical in CBD’s anticancer activity, limiting its therapeutic relevance

#### CB2R agonist

##### Breast cancer

A study (Qamri et al., 2009) reveals that JWH-133 inhibits cell proliferation and migration in breast cancer cell lines (MDA-MB231 and MDA-MB468) through CB2R-dependent pathways. Silencing CB2R using CB2R-specific siRNA reverses these effects, demonstrating the receptor’s critical role. In vivo, JWH-133 reduces tumor growth by 46% and lung metastasis by 76%. JWH-133 also suppresses key pathways linked to cancer progression, such as cyclooxygenase-2 (COX-2) and prostaglandin E2 (PGE2) production, while inhibiting Cdc42 and decreasing the nuclear expression of c-Fos and c-Jun. Furthermore, JWH-133 induces apoptosis by arresting the cell cycle in the G0/G1 phase, evidenced by increased apoptotic markers, and reduces cell proliferation and angiogenesis, as shown by Ki67 and CD31 staining in tumor samples from treated mice.

This in vitro study (Chutoe et al. 2021) investigated the effect of cannabinoid receptor agonists on breast cancer, specifically focusing on the CB2R agonist GW405833. As described in Table 3, GW405833 significantly reduced the viability of MDA-MB-231 breast cancer cells in a concentration-dependent manner, with an IC50 of 23.46 µM. Additionally, GW405833 effectively inhibited cancer cell migration, with a concentration of 15 µM significantly decreasing migration after 24 h of treatment. The study also examined the interaction between breast cancer cells and bone cells using UMR-106 osteoblast-like cells. Pre-treatment of MDA-MB-231 cells with GW405833 before collecting conditioned media significantly improved UMR-106 cell viability by 18.5%, demonstrating the protective role of CB2R activation in reducing the harmful effects of breast cancer cells on bone cells.

**Table 3:** Summary of studies investigating CB2R ligands in cancer cell apoptosis, proliferation, and metastasis

CB2R Agonists					
(Qamri et al. 2009)	JWH-133	Breast cancer	MDA-MB231, MDA-MB468 cell lines	Inhibited COX-2 and PGE2 production, induced apoptosis, G0/G1 cell arrest	Reduce tumor growth by 46%
(Chutoe et al. 2021)	GW405833	Breast Cancer, Bone Metastasis	MDA-MB231, UMR-106 cell lines	Reduced cell viability, induced apoptosis	Suppressed cell proliferation and migration, osteoblast viability by 18.5%
(Blázquez et al. 2008)	JWH-133	Glioblastoma	T98G, U87MG cell lines	Activated mitochondrial apoptotic and autophagic pathways	Inhibited proliferation, induced apoptosis
(Preet et al., 2011)	JWH-015	Non-small cell lung cancer	A549, SW-1573 cell lines	Suppressed AKT signaling, reduced MMP-9 activity	Inhibited migration, metastasis
(Fogli et al. 2006)	JWH-015	Pancreatic cancer	MIA PaCa-2 cell lines	Activated caspase 3/7, inhibited JAK/STAT, MAPK signaling	Induced apoptosis, proliferation
(Brandi et al. 2013)	GW405833	Pancreatic adenocarcinoma	Panc1 cell line	Disrupted Warburg effect, induced autophagy	Inhibited cell proliferation, induced cell death
(Blázquez et al. 2006)	JWH-133	Melanoma	B16, A375, MelJuso, melan-c, and Hermes 2b	Inhibited AKT phosphorylation, causing G1-S cell cycle arrest and apoptosis; reduced tumor vascularization	Significant in melanoma cell proliferation, migration, metastasis, tumor growth, and metastasis to lung and liver
(Duntsch et al. 2006)	KM-233	Glioma	U87MG	Potent cytotoxic effects with IC50 of 1.429 µM; penetrates the blood–brain barrier; minimal toxicity to healthy tissue	Suppressed tumor burden by 50% in SCID mice; superior efficacy compared to BCNU; promising glioma treatment
(Blázquez et al. 2008)	JWH-133	Glioma	C6.9, C6.4, U87 MG and primary glioma cells	Downregulated TIMP-1 expression via the ceramide pathway in cannabinoid-sensitive cells (C6.9, U87 MG, and primary glioma cells). No effect in C6.4 (rat glioma, cannabinoid-resistant))	Reduce tumor growth, invasiveness, and migration in cannabinoid-sensitive cells; no significant effects in cannabinoid-resistant cells (C6.4)
(Jacobsson et al., 2001)	JWH-015	Glioma	Rat C6 glioma cells	Inhibited cAMP levels, suppressed PKA signaling, induced G0/G1 cell cycle arrest; effects reversed by CB2R antagonist SR144528	Significant inhibition of glioma cell proliferation (IC50 = 3.2 µM); confirmed CB2R-mediated antiproliferative effects
	FG158a,	Neuroblastoma	SH-SY5Y, SK-N-BE	Selective CB2R activation (Ki = 21 nM); no CB1R binding; effect reversed by SR144528; downregulated ERK^1/2^ expression	Reduced cell viability in both cell lines; IC50 = 11.8 µM in SH-SY5Y cells

##### Glioblastoma

An in vitro experimental study (Blázquez et al. 2008) investigates how the CB2R agonist JWH133 induces cell death in human glioblastoma cells by activating apoptotic and autophagic pathways. In CB2R-expressing glioblastoma cells T98G and U87MG, JWH133 triggers the mitochondrial apoptotic pathway, leading to mitochondrial membrane potential dissipation and the release of pro-apoptotic factors like cytochrome c, which activates caspases 9 and 3, driving apoptosis. Furthermore, JWH133 induces autophagy, as evidenced by the increased formation of acidic vesicular organelles (AVOs) and the conversion of LC3-I to LC3-II. Silencing autophagy-related genes Atg5 and Atg7 enhances apoptosis, suggesting that autophagy may act as a protective mechanism in CB2R-expressing glioblastoma cells. As highlighted in Table 3, these effects are receptor-specific, with CB2 deficient cells (LN229) showing no response and CB2R antagonists reversing the effects. This emphasizes the therapeutic potential of targeting CB2R in glioblastoma treatment.

##### Non-small cell lung cancer

Extending the focus to non-small cell lung cancer (NSCLC), (Preet et al., 2011) explore CB2R activation using the agonists JWH-015 and JWH-133. In NSCLC cell lines A549 and SW-1573, JWH-015 inhibits cell migration and invasion primarily by suppressing AKT signalling and reducing AKT phosphorylation, critical for cancer cell survival and movement. Additionally, JWH-015 decreases matrix metalloproteinase 9 (MMP-9) expression and activity, a key enzyme involved in metastasis, as confirmed by ELISA and zymography assays*. *In vivo, JWH-133 reduces tumor growth by 70% and lung metastasis by 50% in SCID mice implanted with A549 tumors. These effects are reversed by the CB2R antagonist SR144528, confirming a CB2R-dependent mechanism. Moreover, CB2R activation disrupts focal adhesion formation, reducing vinculin and actin stress fibers, which are crucial for cell migration.

##### Pancreatic cancer

In pancreatic cancer, CB2R activation also shows promise. Research by (Fogli et al. 2006) on MIA PaCa-2 pancreatic cancer cells highlights the cytotoxic effects of the CB2R agonist JWH-015, which induces apoptosis primarily through caspase 3/7 activation. JWH-015 inhibits cell proliferation in a time- and dose-dependent manner while downregulating the JAK/STAT and MAPK signaling pathways, which are crucial for cancer survival. As summarized in Table 3, JWH-015’s ability to reduce cytosolic histone-associated DNA fragments confirms apoptotic cell death, further emphasizing its therapeutic potential. Similarly, another study (Brandi et al. 2013) explores the effects of the CB2R-specific agonist GW405833 (GW) on pancreatic adenocarcinoma cells. Treating Panc1 cells with GW significantly inhibits cell proliferation and induces autophagic cell death by disrupting key cancer-promoting pathways. GW downregulates proteins involved in energy metabolism, such as pyruvate kinase M2 (PKM2) and glyceraldehyde-3-phosphate dehydrogenase (GAPDH), thereby disrupting the Warburg effect. Additionally, GW upregulates mitochondrial matrix protein p32, promoting autophagic cell death by destabilizing mitochondrial membrane potential. GW also leads to the phosphorylation of keratin 18 (KRT18), contributing to cytoskeletal reorganization during cell death, underscoring CB2R activation as a potential therapy for pancreatic cancer.

##### Melanoma

CB2R activation has also shown efficacy in melanoma models. A study by (Blázquez et al. 2006) focusing on the CB2R agonist JWH-133 reports that both human and mouse melanoma cells express CB1Rs and CB2Rs. JWH-133 significantly inhibits melanoma cell proliferation, migration, and metastasis by inhibiting AKT phosphorylation, leading to cell cycle arrest at the G1-S transition and inducing apoptosis. In vivo, JWH-133 reduces tumor growth and lung and liver metastasis in melanoma models, reversing the effects of CB2R antagonism and confirming a CB2R-dependent mechanism. The study also shows that JWH-133 reduces tumor vascularization, as evidenced by reduced CD31 staining and increased TUNEL staining.

##### Glioma

Further emphasizing the promise of CB2R-targeted therapies in glioma, *an *in vitro and in vivo study by (Duntsch et al. 2006) investigates the novel CB2R agonist KM-233. As highlighted in Table 3, KM-233 exhibits potent cytotoxic effects in U87MG glioma cells, with an IC50 value of 1.429 µM, indicating strong efficacy at low concentrations. In vivo, KM-233 significantly reduces tumor burden by 50% in SCID mice, outperforming traditional chemotherapy agents like BCNU. KM-233 penetrates the blood–brain barrier effectively and shows minimal toxicity to healthy brain tissue, making it a strong candidate for glioma treatment.

Further investigation into glioma models reveals the potential for CB2R activation in treating gliomas. A study by (Blázquez et al. 2008) demonstrates that JWH-133 reduces glioma tumor growth and downregulates tissue inhibitor of metalloproteinases-1 (TIMP-1), a protein linked to tumor invasiveness and poor prognosis. JWH-133 reduces TIMP-1 expression via the sphingolipid ceramide pathway, as co-treatment with the ceramide synthesis inhibitor fumonisin B1 reverses the effects. The downregulation of TIMP-1 reduces glioma cell migration, which is associated with decreased tumor proliferation and increased apoptosis.

Finally, in rat C6 glioma cells, a study by (Jacobsson et al., 2001) examines the antiproliferative effects of endogenous and synthetic cannabinoids. JWH-015 significantly inhibits glioma cell proliferation through CB2R activation, with an IC50 of 3.2 µM. This effect is reversed by the CB2R-specific antagonist SR144528, confirming the role of CB2R in mediating these effects. JWH-015 reduces cyclic AMP (cAMP) levels, suppressing protein kinase A (PKA) signaling, leading to G0/G1 cell cycle arrest, and preventing further cell proliferation. Other cannabinoids, such AEA and 2-AG, showed partial reliance on vanilloid receptors, as their effects were attenuated by the vanilloid receptor antagonist capsazepine.

##### Neuroblastoma

A study by (Gado et al. 2022) investigated the anticancer effects of newly synthesized selective CB2R agonists against neuroblastoma cell lines. The research focused on the design and synthesis of compounds based on the 1,8-naphthyridin-2(1H)-one-3-carboxamide scaffold, introducing three ligands, FG158a, FG160a, and FG161a. Structural modifications included bromine, chlorine, or azide substitutions to examine their influence on receptor activity and selectivity. All three compounds exhibited high affinity for CB2R, with Ki values ranging from 16.5 to 45 nM, and no measurable affinity for CB1R (Ki > 10,000 nM), indicating CB2R selectivity.

In vitro, testing on SH-SY5Y and SK-N-BE neuroblastoma cell lines revealed a time and concentration-dependent decrease in cell viability for all compounds. FG158a showed an IC50 of 11.8 µM, FG160a 13.2 µM, and FG161a 27.53 µM in SH-SY5Y cells. Co-treatment with the CB2R antagonist SR144528 partially reversed the cytotoxic effects, supporting a CB2R-mediated mechanism. Additionally, FG158a was found to modulate ERK^1/2^ expression, suggesting possible involvement of the MAPK pathway in its activity. These findings support the relevance of CB2R activation in neuroblastoma and highlight the need for further investigation into these compounds'therapeutic potential.

#### CB2R inverse agonist

##### Pancreatic cancer

AM630 (iodopravadoline), a novel aminoalkylindole, is primarily recognized as an inverse agonist at CB2R in many systems, meaning it not only blocks the receptor but also reduces its basal activity, inducing the opposite effect of typical CB2R activation (Ross et al. 1999). This classification was established (Ross et al. 1999), who demonstrated that AM630 suppresses the constitutive activity of CB2R in certain experimental models. However, despite its inverse agonist properties, AM630 is frequently described as a selective CB2R antagonist in numerous studies, likely because it effectively blocks the actions of CB2R agonists in these settings.

This in vitro study (Fogli et al. 2006) investigates the effects of cannabinoid derivatives on pancreatic cancer MIA PaCa-2 cells, focusing on AM630, a selective CB2R antagonist. AM630 demonstrated moderate cytotoxic effects, with an IC50 of 19.2 ± 1.7 µM after 72 h. Although AM251 was the primary focus, AM630 provided key insights into receptor-independent mechanisms of cannabinoid-induced cytotoxicity. The inhibition of cell growth by AM630 was independent of CB2R activation, as pancreatic cancer cells lacked CB2R expression, suggesting that its cytotoxicity may arise from broader cellular disruptions rather than CB2R antagonism.

Although this study did not fully detail specific pathways involving AM630, cannabinoid treatments were shown to influence critical signaling networks like JAK/STAT and MAPK, which regulate cell proliferation and survival. AM630’s impact on tumor growth may be attributed to interference with these pathways, which contribute to cell death through receptor-independent mechanisms.

#### Melanoma

In an in vitro and in vivo study (Blázquez et al. 2006), summarized in Table 5, examined the role of cannabinoid receptor antagonists and agonists in melanoma treatment, emphasizing AM630. Here, AM630 effectively blocked the antiproliferative effects of cannabinoid receptor agonists, such as WIN-55,212–2, in melanoma cell lines B16 and A375. This confirmed that cannabinoid inhibition of melanoma cell growth was CB2R dependent. AM630 also reversed cannabinoid-induced apoptosis and angiogenesis suppression, demonstrating its ability to counteract the pathways through which cannabinoids slow melanoma progression. Furthermore, AM630 restored phosphorylation of Akt, a survival protein essential for cell cycle progression and melanoma cell viability, further emphasizing its modulation of CB2R-dependent mechanisms.

**Table 4:** Summary of studies investigating CB2R ligands in cancer cell apoptosis, proliferation, and metastasis

CB2R Inverse Agonists					
(Fogli et al. 2006)	AM630	Pancreatic Cancer	MIA PaCa-2 cell line	Cytotoxic effects independent of CB2R activation	Moderate cytotoxicity (IC50 = 19.2 µM), suggesting receptor-independent mechanisms
(Blázquez et al. 2006)	AM630	Melanoma	B16, A375 cell lines	Blocked CB2R agonists'antiproliferative effects, restored Akt signaling	Reversed apoptosis and angiogenesis suppression, demonstrating CB2R-dependent mechanisms
(S. B. Gustafsson et al., 2008)	AM630	Colorectal Carcinoma	Caco-2 cell line	Did not block cannabinoid-induced cytotoxicity, suggesting CB2R independence	Cytotoxicity linked to oxidative stress pathways, further supporting receptor-independent mechanisms

#### Colorectal cancer

A different trend emerged in an in vitro study (S. B. Gustafsson et al., 2008) on the cytotoxic effects of cannabinoids on human colorectal carcinoma cells. Although a CB2R antagonist, AM630 did not mitigate the cytotoxic effects of cannabinoids like HU 210 and AEA in colorectal carcinoma Caco-2 cells. This suggests that the observed cytotoxicity was independent of CB2R activation, as AM630 failed to block cannabinoid-induced reductions in cell viability.

The study on colorectal carcinoma by (S. B. Gustafsson et al., 2008) explored alternative mechanisms, revealing that oxidative stress played a key role in cannabinoid-induced cytotoxicity. Antioxidants like α-tocopherol and the nitric oxide synthase inhibitor L-NAME successfully attenuated the cytotoxic effects, confirming the involvement of oxidative stress pathways. AM630’s inability to block these effects further supported the idea that cannabinoids exert their cytotoxic actions through receptor-independent mechanisms, particularly involving oxidative stress shown in Table 5.

### Synthetic cannabinoids mixed receptor

#### Cannabinoids mixed receptors (CB1/2R) agonist

##### Glioma

Research explored the anticancer actions of synthetic cannabinoids, specifically CP 55,940, WIN 55,212–2, and HU-210, which act as agonists on both CB1 and CB2 receptors. CP 55,940, a synthetic cannabinoid receptor agonist, potently affects CB1 and CB2 receptors. In glioma C6 cells from rats and glioblastoma U373 cells from humans, CP 55,940 activates these receptors, leading to apoptosis and significantly decreasing cell viability (Ortega et al. 2015). CP 55,940 selectively induces cell death in tumor cells through apoptotic mechanisms, sparing primary astrocytes. Another study highlighted its antiproliferative effects in C6 glioma cells, with an IC50 value of 5.6 µM, linked to oxidative stress and ceramide accumulation (Jacobsson et al., 2001). Similarly, HU-210 and CP 55,940 induce cell death in human U373MG glioma cells through CB1R activation but only at high concentrations, raising concerns about their therapeutic potential (Widmer et al. 2008).

WIN 55,212–2, a non-selective cannabinoid receptor agonist, also demonstrates efficacy in glioblastoma models. It induces caspase-independent apoptosis by modulating proteins such as p53 and cathepsin D while reducing HSP70 expression. WIN 55,212–2’s cytotoxic effects suggest its potential as a therapeutic agent against aggressive glioblastomas (Silva et al. 2019).

##### Gastric Cancer

In human gastric adenocarcinoma cell lines (AGS), CP 55,940 induces significant morphological changes, reduces cell viability, and promotes apoptosis through key signaling pathways (Ortega et al. 2016). WIN 55,212–2 also inhibits cell proliferation and invasion in gastric cancer through CB1R activation, inducing apoptosis and reducing phospho-AKT expression (Xian et al. 2010).

##### Breast Cancer

WIN 55,212–2 inhibits cell proliferation and migration in breast cancer by modulating pathways related to cyclooxygenase-2 (COX-2) and prostaglandin E2 (PGE2). It reduces tumor growth and metastasis, with effects reversed by CB1 and CB2 antagonists, confirming the receptor’s role (Qamri et al. 2009). HU-210, however, does not modulate estrogen signaling in breast cancer cells but shows potential therapeutic effects (Okazaki et al. 2016).

##### Colorectal Cancer

HU-210 exerts potent antiproliferative and cytotoxic effects on human colorectal carcinoma Caco-2 cells. Its effects are receptor-independent, as CB1 and CB2 antagonists do not diminish its efficacy. Oxidative stress appears to mediate its cytotoxic action (Widmer et al. 2008).

##### Non-Small Cell Lung Cancer (NSCLC)

WIN 55,212–2 inhibits proliferation and migration in NSCLC cell lines (A549 and SW-1573) by disrupting EGF-induced signaling pathways and attenuating AKT phosphorylation. It effectively reduces tumor growth and increases apoptosis in treated cells (Preet et al., 2011).

##### Melanoma

WIN 55,212–2 disrupts the cell cycle at the G1-S transition, promoting apoptosis while sparing normal melanocytes. It inhibits Akt, a prosurvival protein, and reduces retinoblastoma protein phosphorylation, leading to apoptosis (Blázquez et al. 2006).

##### Mantle Cell Lymphoma (MCL)

WIN 55,212–2 induces ceramide synthase expression (CerS3 and CerS6), enhancing ceramide accumulation through CB1R activation. This promotes apoptosis and reduces cell viability in MCL cell lines (K. Gustafsson et al. 2009).

##### Prostate Cancer

WIN 55,212–2 exhibits cytotoxic effects in prostate cancer cell lines by modulating cannabinoid receptor pathways, emphasizing its therapeutic potential (Silva et al. 2019).

##### Pancreatic Cancer

WIN 55,212–2 shows limited efficacy in MIA PaCa-2 pancreatic cancer cells at 10 µM over 24–72 h, suggesting receptor-independent mechanisms (Fogli et al. 2006).

##### Ewing Sarcoma

WIN-55,212–2 has been identified as a potential target for anticancer drug development in Ewing sarcoma cell lines, specifically TC-71 and A-673. This compound confirmed the presence of CB1R and CB2R mRNA in TC-71 cells, revealing atypical properties of the cannabinoid receptors. Receptor interaction was detected exclusively with the non-selective CB1/CB2R radioligand WIN-55,212–2, not with CP-55,940. This interaction occurred at a single site with high affinity, indicating that the most effective cannabinoids also exhibited a strong affinity for these atypical receptors. Unique signaling profiles were noted, where only cannabinoids with high affinity reduced cell viability and induced cytotoxicity over time. pharmacological profiles and cytotoxic effects were observed in the A-673 Ewing sarcoma cell line (Shoeib et al. 2021).

##### Multiple myeloma

WIN 55,212–2 selectively induces apoptosis in multiple myeloma cell lines via the CB2 receptor, activating caspases and altering Bcl-2 family protein expression, while normal cells remain unaffected. These findings underscore its potential as a therapeutic agent across different cancer types (Barbado et al. 2017).

Overall, synthetic cannabinoids acting on CB1 and CB2 receptors demonstrate significant anticancer activity across various cancer types. Their effects, as summarized in Table 4, include reduced tumor growth, inhibition of proliferation and migration, and increased apoptosis. These findings highlight the therapeutic potential of mixed receptor agonists, though their efficacy and mechanisms vary depending on the cancer type and specific cannabinoids used.

**Table 5:** Summary of studies investigating CB1/CB2R Agonists in cancer cell apoptosis, proliferation, and metastasis

Study	Agonist Used	Cancer Type	Cell Line	Mechanism of Action	Key Outcome
(Ortega et al. 2015)	CP 55,940	Glioma glioblastoma	C6 and U373 cell lines	Activation of Cannabinoid Receptors Induction of Apoptosis Cell Morphological Changes	Cell Viability↓ Anti-proliferative Effects↑ Differential Sensitivity
(Widmer et al. 2008)	CP 55,940	Glioma	U373MG cell line	Activate apoptosis through the activation of downstream effectors, including caspases, which are crucial for programmed cell death	Induces cell death at high concentrations
(Widmer et al. 2008)	HU-210	Glioma	U373MG cell line	Induction of Apoptosis activates the CB1R in U373MG human glioma cells activates ERK and c-Jun NH2-terminal kinase (JNK)	Apoptosis only at high concentrations Cell death, predominantly mediated by CB1R activation
(Silva et al. 2019)	WIN 55,212–2	Glioblastoma	GAMG U251 WI-38 NHA cell lines	Induction of Apoptosis Regulation of Key Proteins Inhibition of Cell Migration Anti-Angiogenic Effects	Selective Cytotoxicity Tumor Growth ↓ Inhibition of Matrix Metalloproteinases (MMPs) Clonogenicity ↓
(Ortega et al. 2016)	CP 55,940	Gastric cancer	AGS cell line	Activates specific signaling pathways associated with apoptosis, involving the regulation of the endocannabinoid system and modulation of kinases induces DNA laddering	Changes in cell morphology ↓ cell viability Apoptotic Induction
(Xian et al. 2010)	WIN 55,212–2	Gastric Cancer	AGS MKN-1 cell lines	Inhibition of Proliferation Induction of Apoptosis Down-Regulation of Phospho-AKT Inhibition of Invasion	In percentages of surviving↓ cells Apoptotic Cell Death In the invasion index of↓ gastric cancer cells
(Qamri et al., 2009)	WIN 55,212–2	Breast cancer	MDA-MB231, MDA- MB231-luc, and MDA-MB468 cell lines	Inhibition of Cell Proliferation Inhibition of Migration Modulation of Signaling Pathways	Tumor Growth ↓ Metastasis Inhibition
(Okazaki et al. 2016)	HU-210	Breast cancer	MCF-7 cell line	Does not modulate estrogen receptor (ER) signaling in MCF-7 human breast cancer cells affects cell morphology and viability in a concentration-dependent manner	No Anti-Estrogenic Activity Lacks the endocrine-disrupting effects
(Widmer et al. 2008)	HU-210	Colorectal carcinoma	Caco-2 cell line	The cytotoxic effects were suggested to involve oxidative stress synergistic cytotoxic effects when combined with the chemotherapeutic agent 5-fluorouracil	Cytotoxic Effects Oxidative stress
(Preet et al., 2011)	WIN 55,212–2	Non–small cell lung cancer	A549 and SW-1573 cell lines	Inhibition of Cell Proliferation Reduced Migration and Invasion Inhibition of AKT Phosphorylation Decreased MMP-9 Activity	Inhibition of Tumor Growth In Lung Metastasis↓ Apoptosis↑
(Blázquez et al. 2006)	WIN 55,212–2	Melanoma	B16 A375 MelJuso	Inhibition of Cell Growth Cell Cycle Arrest Induction of Apoptosis	Tumor Growth↓ Metastatic Spread ↓ Apoptosis↑
(K. Gustafsson et al. 2009)	WIN 55,212–2	Mantle cell lymphoma	Rec-1 mantle cell lymphoma cell lines	Activation of CB1 Receptors Induction of Ceramide Synthases Ceramide Accumulation	Apoptosis ↑ Ceramide Species Induction
(Silva et al. 2019)	WIN 55,212–2	Prostate cancer cells	GAMG U251 WI-38 NHA cell lines	Induction of Apoptosis Regulation of Key Proteins Inhibition of Cell Migration Anti-Angiogenic Effects	Selective Cytotoxicity Tumor Growth ↓ Inhibition of Matrix Metalloproteinases (MMPs) Clonogenicity ↓
(Fogli et al. 2006)	WIN 55,212–2	Pancreatic cancer	MIA PaCa-2 cell line	cannabinoid-mediated cytotoxic effects	Did not significantly affect cell viability
(Shoeib et al. 2021)	WIN 55,212–2	Ewing sarcoma	TC-71 and A-673 cell lines	G-Protein Activation Distinct Signaling Profiles	Cytotoxicity Cell Viability↓ The cannabinoid receptors expressed in EWS cells exhibit atypical properties compared to canonical cannabinoid receptors, resulting in unique functional profiles
(Shoeib et al. 2021)	CP 55,940	Ewing sarcoma	TC-71 and A-673 cell lines	inhibition of adenylyl cyclase activity by CB1R and CB2R	↓cell viability and induce↓ cytotoxicity in a time-dependent manner
(Barbado et al. 2017)	WIN 55,212–2	Multiple myeloma	U266 RPMI8226 MM1.S U266-LR7 RPMI8226-LR5 MM1.R cell lines	Induction of Apoptosis Caspase Activation Alteration of Bcl-2 Family Proteins Ceramide Accumulation Modulation of Signaling Pathways	Tumor Growth↓ Metastatic Spread↓ Apoptosis↑

#### Cannabinoids mixed receptors (cb1/2r) inverse agonist and antagonist

There are currently no studies available that specifically examine agents functioning as mixed antagonists or inverse agonists for cannabinoid receptors CB1 and CB2.

## Discussion

This systematic review examines the mode of action of synthetic cannabinoids and their impact on the cannabinoid receptors. Ultimately, discovering the mechanisms by which these synthetic agents could modulate the cancer progression or reduction. Cannabis receptors are presented in the cancerous cells in various percentages, enabling these receptors to be targeted as possible therapeutic agents (De Jesús et al., 2010) (X. Xu et al., 2006). Moreover, these receptors demonstrated noticeable cancerous cells modulating effect via multiple mechanisms such as enhancing apoptosis, reducing the angiogenesis, or lowering the cancer metastasis (Pennant & Hinton, 2023). All these proposed mechanisms modulating cancer cells using synthetic cannabinoid agents are evaluated in this systematic review, providing a comprehensive overview of the available synthetic cannabinoids and their potential therapeutic effects. Furthermore, investigate how the mode of action modulates the synthetic cannabinoids effects.

### Synthetic cannabinoids 1 receptor

The findings indicate that CB1R antagonists are used in conjunction with CB2R agonists to enhance the anticancer effects of CB2R or to highlight that the anticancer activity is primarily mediated by CB2R agonists. Research on CB1R agonists is quite limited, with only one study focusing on their effects on cancerous cells (Ellert-Miklaszewska, 2021). This study investigated the human glioblastoma multiforme (GBM) cell line U87MG in both in vivo and in vitro settings, showing a reduction in tumor growth (Gurley et al., 2012). Therefore, further research is needed to confirm the role of CB1R agonists in cancer therapy. Additionally, there is a lack of studies examining the effectiveness of CB1R inverse agonists in cancer modulation. As a result, more investigations are necessary to explore the role of these synthetic cannabinoids and their interactions with CB1R, including the mechanisms of agonists and inverse agonists, to enhance our understanding of their potential applications.

### Synthetic Cannabinoids 2 Receptor

These findings suggest that CB2R agonists are the main modulators of cancer cell reduction among various cancerous cell lines with multiple proposed mechanisms. Furthermore, CB2R could be a promising target for treating many cancer types via agonistic activity. One example of this agonistic activity on CB2R is JWH-133, which is a selective agonist for CB2R. This agent inhibits the growth and survival of cancerous cells (S. Xu et al., 2019). Meanwhile, CB1R antagonists are involved in various studies in combination with CB2R agonists either to validate the effect of CB2R agonists by blocking the CB1R or to potentiate the CB2R agonist function to reduce tumour growth. The role of the CR1R antagonist emphasizes the vital role of CB2R agonists.

For CB2R antagonists, studies focus on its role in blocking the cancer-fighting effects of cannabinoids that target the CB2R. For example, SR144528 was found to reverse the effects of JWH-133 in breast cancer research, which normally slows down cancer cell growth and migration. By applying SR144528, researchers could confirm that these effects depended on CB2 receptors. Other research papers studied the same concept with several CB2R antagonists. On the other hand, studies involving inverse agonist acts on CB2R showed a moderate ability to kill cancerous cells depending on the cancer type.

Furthermore, there are many mechanisms by which CB2R inverse agonists act on cancerous cells, including blocking pathways through which cancer cells can survive. Another mechanism unrelated to CB2R is shown in the results. All in all, antagonist acts on CB2R were mainly utilized to validate the impact of CB2R receptor cancer cell-mediated reduction. In contrast, the inverse agonist showed possible minor effects on cancer cell development and growth.

### Synthetic cannabinoids mixed receptor

CBRs agonists, such as CP 55,940, WIN 55,212–2, and HU-210, demonstrate potential as anticancer agents by activating CB1 and CB2 receptors. CP 55,940 has shown strong efficacy in inducing apoptosis and decreasing cell viability in glioma and gastric cancer cells through the activation of these receptors and modulation of key signaling pathways. Consequently, it effectively alters cell density, highlighting its therapeutic potential for glial tumors. Further studies are necessary to elucidate the mechanisms of action for mixed CBR antagonists and inverse agonists. While some of these compounds show promising anticancer effects, more research is needed to fully understand their mechanisms of action.

### Therapeutic implications

These findings highlight the potential of synthetic cannabinoids as promising anticancer agents, particularly due to their ability to target specific pathways selectively. This selectivity may allow for the development of treatments that avoid the adverse effects commonly associated with traditional chemotherapy by sparing healthy cells. Moreover, synthetic cannabinoids offer the potential to overcome resistance mechanisms seen in some cancer therapies by acting through unique pathways. These include inducing apoptosis specifically in cancer cells via CB2R activation, inhibiting cell proliferation through pathways like PI3 K/AKT and MAPK, reducing angiogenesis to limit tumor growth, modulating the tumor microenvironment to enhance immune detection, and lowering inflammation associated with cancer progression. As our understanding of cannabinoid receptor signaling grows, it opens opportunities for tailored treatments based on individual tumor characteristics, potentially leading to more personalized and effective cancer therapies.

### Limitations and future directions

One significant limitation of this systematic review is the relatively small sample size and narrow focus of the included studies, which may impact the generalizability of these findings across various cancer types and patient populations. Additionally, inconsistencies in methodologies, such as variations in cannabinoid dosages, types, and treatment durations, pose challenges for direct comparisons and may undermine the reliability of the results. Furthermore, as most of the studies were conducted in vitro, there are inherent limitations in applying these findings to in vivo environments, where the responses of cell lines may not fully reflect the complex interactions present in living organisms. The lack of long-term studies also restricts our understanding of the sustained efficacy and potential adverse effects associated with synthetic cannabinoids.Moreover, many novel modes of action as allosteric modulation and bitopic ligands, have not been fully understood in the context of cancer, which limits the systematic review scope to the agonist, antagonist, and inverse agonist as modes of action. These limitations emphasize the need for more rigorously designed research across diverse models and populations to gain a clearer understanding of the therapeutic potential and safety of synthetic cannabinoids in cancer treatment.

## Conclusion

The current systematic review introduces the anti-tumor mechanisms of synthetic cannabinoid receptors'ligands and their underlying mechanism of action. CB1R agonist illustrated a higher possibility of cellular signalling pathways alteration for cancer pathways, providing an optional cancer treatment. However, the CB1R antagonist and inverse agonist were utilized as confirmatory to CB2R agonistic activity.

Moreover, the research on CB2R antagonists and agonists highlighted their crucial roles in mediating cancer models'antiproliferative, antimetastatic, and cytotoxic effects. CB2R-specific antagonists reverse the anticancer effects of CB2R agonists, underscoring CB2R involvement in tumour growth inhibition and metastasis. Studies across various cancer types, including breast, glioblastoma, non-small cell lung, and pancreatic cancer, reveal that CB2R activation via agonists triggers apoptosis, autophagy, and significant reductions in cell proliferation, migration, and angiogenesis. In contrast, some studies that revealed the role of CB2R antagonists showed mixed results, often blocking the CB2R-mediated effects but also exhibiting cytotoxicity in a receptor-independent manner in certain cancers.

Synthetic cannabinoids show significant potential as therapeutic agents by targeting both CB1 and CB2 as receptor agonists to reduce tumour growth and promote apoptosis across various cancers. Their diverse mechanisms, including modulation of signalling pathways and oxidative stress, underline their promise in treatment. However, the absence of research on these receptors’ mixed antagonists or inverse agonists highlights a critical gap, suggesting a valuable direction for future studies to enhance our understanding of cannabinoid signalling in cancer therapy.

## Data Availability

No datasets were generated or analysed during the current study.

## Abbreviations

AEA: N-arachidonoyl-ethanolamine
2-AG: 2-Arachidonoylglycerol
CBR: Cannabinoid Receptor
CB1R: Cannabinoid Receptor 1
CB2R: Cannabinoid Receptor 2
CINP: Chemotherapy-Induced Neuropathic Pain
ECS: Endocannabinoid System
ERK: Extracellular Signal-Regulated Kinase
GCPR: G Protein-Coupled Receptor
IL: Interleukin
IL-6: Interleukin 6
IL-10: Interleukin 10
MMP: Matrix Metalloproteinase
NSCLC: Non-Small Cell Lung Cancer
PKA: Protein Kinase A
THC: Tetrahydrocannabinol
TIMP: Tissue Inhibitor of Metalloproteinases
CNS: Central Nervous System
PGE2: Prostaglandin E2
JAK: Janus Kinase
CVD: Cardiovascular Disease
G1/S: G1 to S Phase Transition (cell cycle)
Ki: Inhibition Constant
AVOs: Acidic Vesicular Organelles
Cdc42: Cell Division Control Protein 42
DAPI: 4',6-Diamidino-2-Phenylindole (a fluorescent stain)
ERK1/2: Extracellular Signal-Regulated Kinase 1 and 2
P38: P38 Mitogen-Activated Protein Kinase
5-FU: 5-Fluorouracil
C6: C6 Glioma Cell Line
MDA-MB: Michigan Cancer Foundation-7 Breast Cancer Cell Line
RKO: RKO Colon Carcinoma Cell Line
SW480: SW480 Colorectal Cancer Cell Line
HT29: HT29 Colorectal Cancer Cell Line
DLD-1: DLD-1 Colorectal Cancer Cell Line
HCT116: HCT116 Colorectal Cancer Cell Line
LS174: LS174T Colorectal Carcinoma Cell Line
A549: A549 Lung Carcinoma Cell Line
MIA PaCa-2: MIA PaCa-2 Pancreatic Cancer Cell Line
U373MG: U373MG Human Glioma Cell Line
B16: B16 Melanoma Cell Line
A375: A375 Melanoma Cell Line
T47D: T47D Breast Cancer Cell Line
MCF-7: Michigan Cancer Foundation-7 Breast Cancer Cell Line
AM251: A selective CB1R antagonist
AM630: A selective CB2R antagonist
SR141716: Rimonabant, a selective CB1R antagonist
SR144528: A selective CB2R antagonist
JWH-133: A selective CB2R agonist
JWH-015: Another selective CB2R agonist
WIN 55,212–2: A non-selective cannabinoid receptor agonist
MDA-MB231: A specific breast cancer cell line
Caco-2: Human colorectal adenocarcinoma cell line
SW-1573: Non-small cell lung cancer cell line
U118: U118 Human Glioma Cell Line
U87: U87 Human Glioblastoma Cell Line
C57BL/6: A common laboratory mouse strain
p38 MAPK: P38 Mitogen-Activated Protein Kinase
ERK MAPK: Extracellular Signal-Regulated Kinase Mitogen-Activated Protein Kinase
JTE 907: A selective inverse agonist of CB2R
HU 308: A synthetic cannabinoid that selectively binds to CB2R
PTI: Pharmacological Testing of Inhibitors
AP-1: Activator Protein 1
NF-κB: Nuclear Factor kappa-light-chain-enhancer of activated B cells
TGF-β: Transforming Growth Factor Beta
Nrf2: Nuclear factor erythroid 2-related factor 2
ROS: Reactive Oxygen Species
DNA: Deoxyribonucleic Acid
RNA: Ribonucleic Acid
PCR: Polymerase Chain Reaction
FBS: Fetal Bovine Serum
DMSO: Dimethyl Sulfoxide
MTT: 3-(4,5-Dimethylthiazol-2-yl)-2,5-diphenyltetrazolium bromide
IC50: Half Maximal Inhibitory Concentration
LDH: Lactate Dehydrogenase
ATP: Adenosine Triphosphate
MAPK: Mitogen-Activated Protein Kinase
HIF-1α: Hypoxia-Inducible Factor 1-alpha
CXCL: C-X-C Motif Chemokine Ligand
PDGF: Platelet-Derived Growth Factor
VEGF: Vascular Endothelial Growth Factor
SDF-1: Stromal Cell-Derived Factor 1
TAM: Tumor-Associated Macrophage
NFAT: Nuclear Factor of Activated T-cells
cAMP: Cyclic Adenosine Monophosphate
ER: Estrogen Receptor
PR: Progesterone Receptor
SOD: Superoxide Dismutase
NADPH: Nicotinamide Adenine Dinucleotide Phosphate
AMPK: AMP-Activated Protein Kinase
HSP: Heat Shock Protein
IFN: Interferon
LPS: Lipopolysaccharide
TAM: Tamoxifen
Treg: Regulatory T Cell
WHO: World Health Organization
gonist: Inverse Agonist
